# Supplementary material for: A mitochondria-anchored isoform of the actin-nucleating spire protein regulates mitochondrial division
Source: eLife. 2015 Aug 25;4:e08828. doi: 10.7554/eLife.08828 (PMC4574297; doi:10.7554/eLife.08828)
Supplement: Figure 8—source data 1. — DOI: http://dx.doi.org/10.7554/eLife.08828.020 [file elife08828s001.docx]

|  | Less constricted | Average constricted | More constricted |
| --- | --- | --- | --- |
| L [nm] | 680 | 680 | 680 |
| R [nm] | 230 | 230 | 230 |
| r [nm] | 145 | 110 | 65 |
| r/R | 0.63 | 0.48 | 0.28 |
| pressure [Pa] | 271.28 | 388.29 | 674.32 |
| actin filaments surface density [1/μm2] | 271.28 | 388.29 | 674.32 |
| distance between actin filament ends [nm] | 60.71 | 50.74 | 38.51 |
| Total number of filaments | 14.83 | 16.1 | 16.52 |
| line tension [pN] | 2.36 | 2.56 | 2.63 |
